# Machine Learning Prediction Models to Reduce Length of Stay at Ambulatory Surgery Centers Through Case Resequencing

**DOI:** 10.1007/s10916-023-01966-9

**Published:** 2023-07-10

**Authors:** Jeffrey L. Tully, William Zhong, Sierra Simpson, Brian P. Curran, Alvaro A. Macias, Ruth S. Waterman, Rodney A. Gabriel

**Affiliations:** 1 Department of Anesthesiology, Division of Perioperative Informatics, University of California, San Diego, La Jolla, CA USA; 2 Department of Medicine, Division of Biomedical Informatics, University of California, San Diego, La Jolla, CA USA

**Keywords:** Perioperative resource management, Outpatient surgery, Machine learning, Artificial intelligence, Perioperative informatics

## Abstract

The post-anesthesia care unit (PACU) length of stay is an important perioperative efficiency metric. The aim of this study was to develop machine learning models to predict ambulatory surgery patients at risk for prolonged PACU length of stay - using only pre-operatively identified factors - and then to simulate the effectiveness in reducing the need for after-hours PACU staffing. Several machine learning classifier models were built to predict prolonged PACU length of stay (defined as PACU stay ≥ 3 hours) on a training set. A case resequencing exercise was then performed on the test set, in which historic cases were re-sequenced based on the predicted risk for prolonged PACU length of stay. The frequency of patients remaining in the PACU after-hours (≥ 7:00 pm) were compared between the simulated operating days versus actual operating room days. There were 10,928 ambulatory surgical patients included in the analysis, of which 580 (5.31%) had a PACU length of stay ≥ 3 hours. XGBoost with SMOTE performed the best (AUC = 0.712). The case resequencing exercise utilizing the XGBoost model resulted in an over three-fold improvement in the number of days in which patients would be in the PACU past 7pm as compared with historic performance (41% versus 12%, P<0.0001). Predictive models using preoperative patient characteristics may allow for optimized case sequencing, which may mitigate the effects of prolonged PACU lengths of stay on after-hours staffing utilization.

**Supplementary Information:**

The online version contains supplementary material available at 10.1007/s10916-023-01966-9.

## Introduction

Post-anesthesia care unit (PACU) length of stay (LOS) is an important focus of efforts to improve quality and decrease costs of perioperative care, particularly in the outpatient surgery center where patient throughput is a key determinant of efficiency and related financial metrics [1, 2]. The issues associated with prolonged PACU stay (especially when the stay occurs after-hours in a freestanding ambulatory surgery center) include increased risk for hospital admission, decreased patient satisfaction, and increased staffing and operational costs [3–8]. Optimizing the sequencing of surgical case order in an operating room may aid in reducing PACU usage after-hours (e.g. patients predicted to have the longest PACU stays could be rescheduled to occur earlier in the day).

The development of predictive models for prolonged PACU LOS could be clinically useful in the optimization of case order sequencing with the goal of reducing after-hours PACU stay in an ambulatory surgery center. Analysis of perioperative data with machine learning techniques have been used for the development of predictive models aimed at improving PACU efficiency [9, 10]. Previously, we reported the development of a logistic regression-based predictive model for prolonged PACU LOS after outpatient surgery [10]. In this current study, the objective was to develop various machine learning models aimed at identifying patients at risk for prolonged PACU LOS and then utilize the models to optimize case sequencing in a simulation. We hypothesized that by optimizing operating room case sequencing based on predicted risk for prolonged PACU LOS, we could reduce the frequency of patients required to remain in the PACU after-hours.

## Methods

### Study sample

As the dataset did not contain any identifiers or other protected health information it was deemed exempt from informed consent requirements by our Institutional Review Board. This report adheres to the SQUIRE guidelines for quality improvement studies [11]. Data was retrospectively obtained from procedures occurring at our institution’s standalone outpatient surgery center between March of 2018 and November of 2020. The objectives of this study were to: 1) develop a predictive model using preoperative features to classify patients that were high risk for prolonged PACU stay; and 2) utilize this model to simulate case re-sequencing (in which patients higher at risk for prolonged PACU stay were scheduled earlier in the day) on historic data and compare frequency of after-hour staffing needs.

For the predictive model, the primary dependent variable was a binary variable that classified prolonged PACU LOS, defined as ≥ 3 hours (0 = PACU stay was < 3 hours and 1 = PACU stay was ≥ 3 hours). This threshold was chosen as it represented the 75% quartile of PACU stay duration. The following independent features were obtained for each case: surgical procedure (Supplementary Table 1 lists frequency of each surgical procedure), American Society of Anesthesiologists (ASA) Physical Status (PS) Classification, sex, age, scheduled case duration in minutes, and body mass index (BMI). Additionally, selected comorbidities were identified based on International Classification of Diseases, Ninth and Tenth Revision Codes (ICD9, ICD10, respectively). All ICD9 and ICD10 codes assigned prior to day of surgery were collected. The diagnosis with the highest frequencies among the entire dataset were then included as features. These included patients identified as active smokers or having history of alcohol abuse, anxiety, asthma, chronic kidney disease (CKD), chronic obstructive pulmonary disease (COPD), chronic pain, coronary artery disease (CAD), depression, diabetes mellitus (DM), dysrhythmias, gastroesophageal reflux disease (GERD), history of seizures, hypertension, hypothyroidism, or obstructive sleep apnea (OSA). Patients who had multiple surgical encounters had each encounter treated as a unique patient case.

### Statistical analysis

Python (v3.10.4) was used for all statistical analysis. First, the data was divided into two datasets, a training dataset and a blind test dataset, utilizing an 80:20 split respectively using a randomized splitter— the “train_test_split” method from the sci-kit learn library—thus, proportions for the binary outcome stayed roughly the same in both datasets [12]. K-fold cross validation was used on the training dataset to optimize each machine learning model (measuring sensitivity, specificity, and area under the curve (AUC) for receiver operating characteristics curve). The models with the optimal hyperparameters were then tested on the blind test dataset. The area under the curve (AUC) for receiver operating characteristics curve were measured for each model after being implemented on the blind test set to evaluate model performance. Calibration curves were also developed to examine the fit of the model on the blind test dataset with the top performing model. The predicted risk was plotted against the observed risk for each of the 10 risk percentiles created from the data set.

### Data balancing

Synthetic Minority Oversampling Technique (SMOTE) and random under-sampling were both implemented using the “imblearn” library to obtain a balanced class distribution with minimal differences between positive and negative outcomes. Datasets with large differences in positive and negative outcomes were considered to be unbalanced. Imbalance data may make it difficult for predictive modeling due given the uneven distribution of positive and negative outcomes. Random under-sampling of the majority class is frequently used to obtain a more balanced class distribution. SMOTE is a technique that oversamples the minority class to reduce the impact of data imbalance—without affecting the majority class [13]. SMOTE takes samples from the target class and five of its nearest neighbors, and then generates synthetic samples, increasing the percentage of the target class. Combining random under-sampling and SMOTE improved the model outcomes. Both techniques were only applied to the training splits. The SMOTE and random under-sampling ratios for each model were optimized using k-folds cross validation on the training dataset. SMOTE and random under-sampling were found to be more successful than solely using SMOTE or random under-sampling.

### Machine learning models

We evaluated six different classification models: logistic regression, feedforward neural network, XGBoost regressor, balanced random forest classifier, balanced bagging classifier, and random forest classifier. For each model, we compared the following: oversampling the training set via SMOTE and no oversampling or undersampling technique. We report results using no oversampling or undersampling technique and results from using both. For each model, all features were included as inputs. One-hot encoding was used for categorical features. For each machine learning model, we performed hyperparameter tuning based on k-folds cross validation prior to performing the final version on that model.

The logistic regression classifier predicts probabilities for each of the different class possibilities based on the model input. A limited-memory BFGS (L-BFGS) solver was implemented without specifying individual class weights. This model provided a baseline score and helped make the case for improvement over the evaluation metrics. The optimal value for C (inverse of regularization strength) was found to be 6. The optimal SMOTE ratio was found to be 0.75. The optimal RandomUnderSampler ratio was found to be 0.9.

Using the Keras interface for Tensorflow [14], we built a basic shallow feed-forward neural network. The activation function was set to the rectified linear unit function. For hyperparameter tuning, we performed grid search cross-validation to find the optimal parameter values for number of hidden layers, number of neurons per hidden layer, maximum number of iterations, batch size, and learning rate, which were 1, 128, 100, 64, and 0.0001 respectively. The optimal SMOTE ratio was found to be 0.4. The optimal RandomUnderSampler ratio was found to be 0.5.eXtreme Gradient Boosting or XGBoost is an optimized version of the tree boosting system [15]. After hyperparameter tuning, we found the optimal parameter values for the objective, subsample ratio of columns when constructing each tree, learning rate, maximum depth of a tree, number of estimators, and L2 regularization term on weights, to be binary:hinge, 0.55, 0.01, 300, 500, and 1.5, respectively. The optimal SMOTE ratio was found to be 0.5. The optimal RandomUnderSampler ratio was found to be 0.85.

Random forest is a technique that combines the predictions from multiple decision trees together to make more accurate predictions than an individual tree [16]. After hyperparameter tuning, we found the optimal parameter value for the number of estimators to be 500. The optimal SMOTE ratio was found to be 0.8. The optimal RandomUnderSampler ratio was found to be 0.9. The Balanced Random Forest is an implementation of the random forest, which randomly under-samples each bootstrap to balance it. After hyperparameter tuning, we found the optimal parameter values for the number of estimators and sampling_strategy (the desired ratio, after resampling, of the number of the minority class over the number of the majority class) to be 2000 and 0.95 respectively. The optimal SMOTE ratio was found to be 0.35. The optimal RandomUnderSampler ratio was found to be 0.75. Bagging or bootstrap-aggregating is another way to develop ensemble models. Bagging methods build several models on random subsets of the original dataset. The predictions are then aggregated to form a final prediction. Bagging classifiers are generally more immune to overfitting. After hyperparameter tuning, we found the optimal parameter value for the number of estimators to be 1200. The optimal SMOTE ratio was found to be 0.4. The optimal RandomUnderSampler ratio was found to be 0.6.

### SHAP analysis

SHapley Additive exPlanations or SHAP was used to determine feature importance for the highest performing model [17]. Frequently, machine learning models can be hard to interpret. SHAP provides a framework to interpret the predictions of a complex machine learning model by giving each input feature an importance value for a specific prediction.

### Case resequencing

**Fig. 1:**
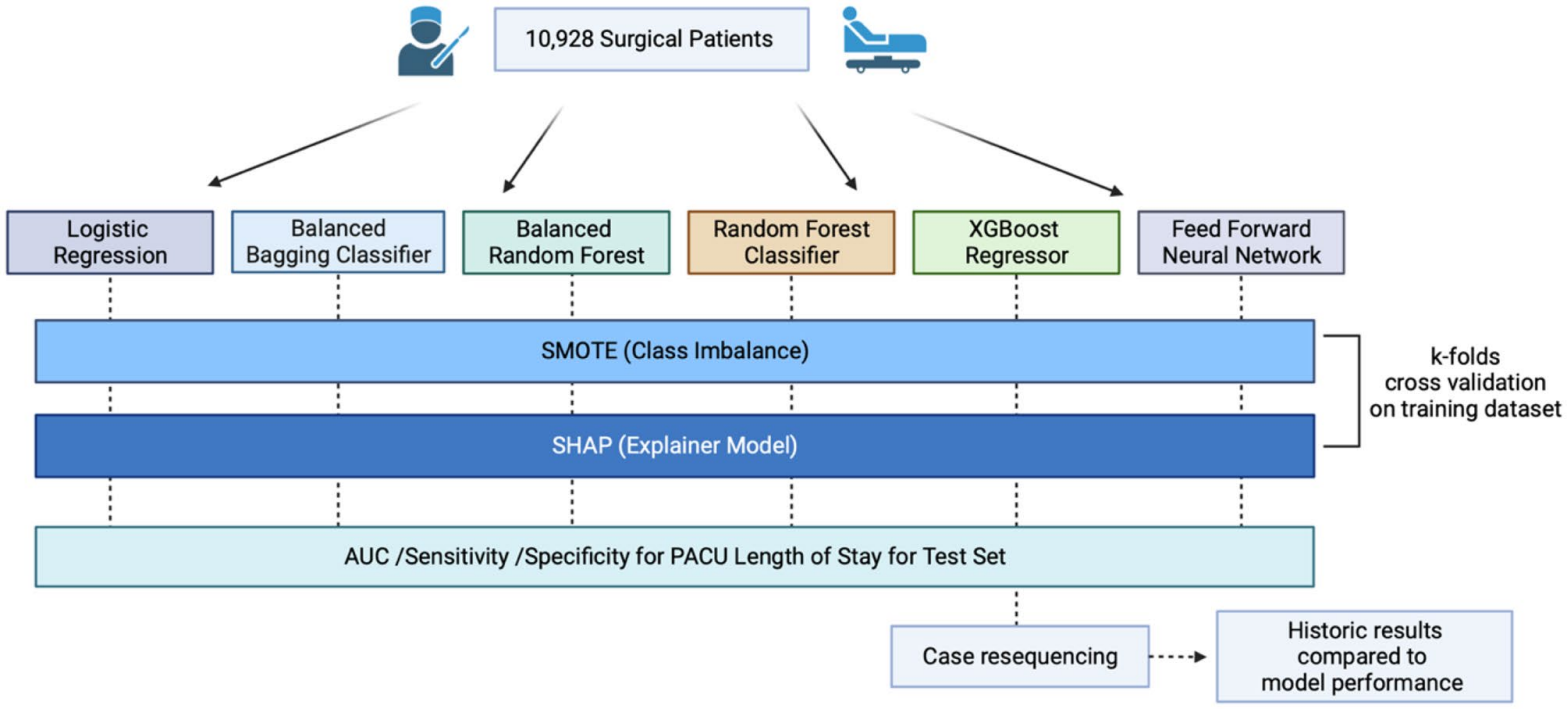
Study methodology

After performance evaluation of each classification model was complete, the highest performing model was then used in an exercise to re-sequence case order against historic results. The re-sequencing was performed on the test set (20% of entire dataset), which consisted of 198 previous days in which a full OR day (defined as cases scheduled at least past 3pm) was scheduled. The model was then used to predict which cases would have a prolonged PACU LOS. Identified cases with the highest probability of prolonged PACU LOS were scheduled earliest, while those with lowest probability were scheduled later. One hundred and sixty-two cases were resequenced and the historic results were compared against model performance. The chi-square test was used to compare frequencies between the simulated versus actual historic cohorts’ number of times patients were in the PACU past 7pm (Fig. 1).

## Results

The final study population consisted of 10,928 patient cases, in which 580 (5.31%) had PACU LOS ≥ 3 hours (Table 1). The median [quartile] PACU LOS in the non-prolonged PACU stay versus prolonged PACU stay cohorts were 81 [61, 107] minutes versus 210 [192, 246] minutes, respectively. On unadjusted analyses, female sex (P<0.0001) and scheduled surgical case duration (P<0.0001) were associated with prolonged PACU LOS.

**Table 1:** Differences in patient and surgical characteristics between the two outpatient surgery cohorts (PACU LOS < 3 hours versus PACU LOS ≥ 3 hours)

	**PACU LOS < 3 h**		**PACU LOS ≥ 3 h**		
	**n**	**%**	**n**	**%**	**P-value**
Total	10,348		580		
PACU LOS (minutes), median [quartile]	81 [62, 107]		210 [192, 246]		< 0.0001
ASA PS Score					0.14
1	1995	19.3	97	16.7	
2	17	0.2	1	0.2	
3	5879	56.8	358	61.7	
4	2457	23.7	124	21.4	
Age (years), median [quartile]	51 [37, 63]		50 [39, 63]		0.98
Male sex	4064	39.3	130	22.4	< 0.0001
Scheduled Case Duration (minutes), median [quartile]	60 [38, 95]		85 [55, 140]		< 0.0001
Body Mass Index (kg/m2), median [quartile]	26.5 [23.3, 30.6]		26.7 [23.3, 30.0]		0.55
Comorbidities					
Active Smoker	252	2.4	8	1.4	0.14
Alcohol Abuse History	574	5.5	6	1.0	0.06
Anxiety	942	9.1	51	8.8	0.86
Asthma	568	5.5	12	2.1	0.09
Chronic Kidney Disease	66	0.6	3	0.5	0.93
Chronic Obstructive Pulmonary Disease	68	0.7	2	0.3	0.52
Chronic Pain	253	2.4	20	3.4	0.17
Coronary Artery Disease	158	1.5	4	0.7	0.15
Depression	676	6.5	46	7.9	0.22
Diabetes Mellitus	171	1.7	15	2.6	0.13
Dysrhythmias	195	1.9	13	2.2	0.65
Gastroesophageal Reflux Disease	959	9.3	37	6.4	0.03
History of Seizures	76	0.7	3	0.5	0.73
Hypertension	1561	15.1	82	14.1	0.57
Hypothyroidism	606	5.9	25	4.3	0.14
Obstructive Sleep Apnea	550	5.3	30	5.2	0.44

*ASAPS* American Society of Anesthesiologists Physical Status, *LOS* Length of Stay, *PACU* Post-Anesthesia Care Unit

Each machine learning model was trained on the training set (80% of original dataset). Using 10-fold cross-validation on the training set, hyperparameters for each model type were optimized (Table 2) before they were then validated on the separate test set

**Table 2:** Performance metrics of all models with their optimal hyperparameters based on k-folds cross-validation

**Classification Model**	**AUC (95% CI)**	**AUC (95% CI)** **(SMOT)**	**Specificity (95% CI)**	**Specificity (95% CI)** **(SMOT)**	**Sensitivity (95% CI)**	**Sensitivity (95% CI)** **(SMOT)**
Neural Network	0.586 (0.557, 0.615)	0.628 (0.583, 0.673)	0.969 (0.943, 0.994)	0.880 (0.835, 0.925)	0.204 (0.137, 0.271)	0.375 (0.282, 0.467)
XGBoost	0.663 (0.624, 0.702)	0.685 (0.652, 0.718)	0.964 (0.948, 0.979)	0.917 (0.905, 0.929)	0.363 (0.287, 0.439)	0.452 (0.387, 0.517)
Random Forest Classifier	0.625 (0.584, 0.666)	0.637 (0.600, 0.676)	0.969 (0.965, 0.973)	0.952 (0.932, 0.972)	0.279 (0.197, 0.361)	0.209 (0.156, 0.262)
Logistic Regression	0.589 (0.560, 0.618)	0.667 (0.628, 0.706)	0.971 (0.961, 0.980)	0.744 (0.704, 0.783)	0.209 (0.156, 0.262)	0.591 (0.526, 0.656)
Balanced Bagging Classifier	0.672 (0.627, 0.717)	0.657 (0.624, 0.690)	0.814 (0.784, 0.843)	0.858 (0.842, 0.874)	0.529 (0.456, 0.602)	0.457 (0.396, 0.518)
Balanced Random Forest Classifier	0.684 (0.653, 0.715)	0.681 (0.642, 0.720)	0.727 (0.709, 0.744)	0.819 (0.792, 0.846)	0.642 (0.577, 0.707)	0.542 (0.466, 0.618)

*AUC* Area under the receiver operating characteristics curve, *CI* Confidence Interval, *SMOTE* Synthetic Minority Oversampling Technique, bolded numbers indicate best performance for that metric

**Fig. 2:**
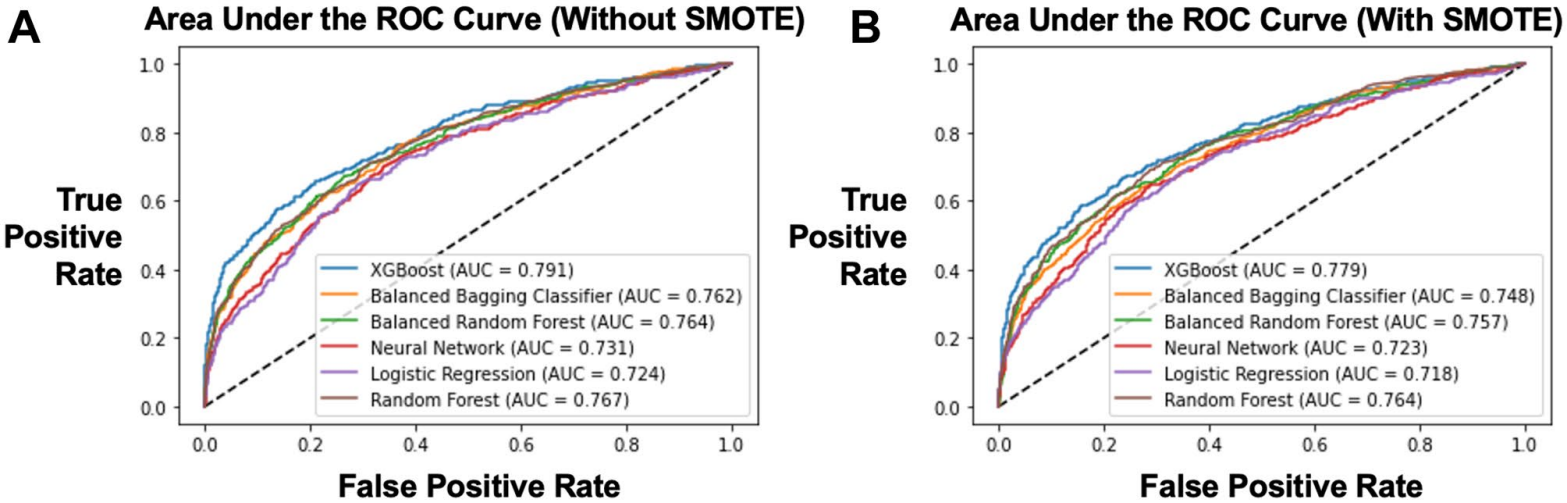
Comparison of Machine Learning Model Performance Via Area Under the Receiver Operating Characteristic (ROC) Curve With and Without Synthetic Minority Oversampling Technique (SMOTE)

The models were then tested on the hold out test set. For each model, performance was compared when SMOTE was versus not used. Based on AUC, the best performing model with SMOTE was XGBoost (AUC 0.779), whereas the worst performing model with SMOTE was logistic regression without SMOTE (AUC 0.718) (Fig. 2).

**Fig. 3:**
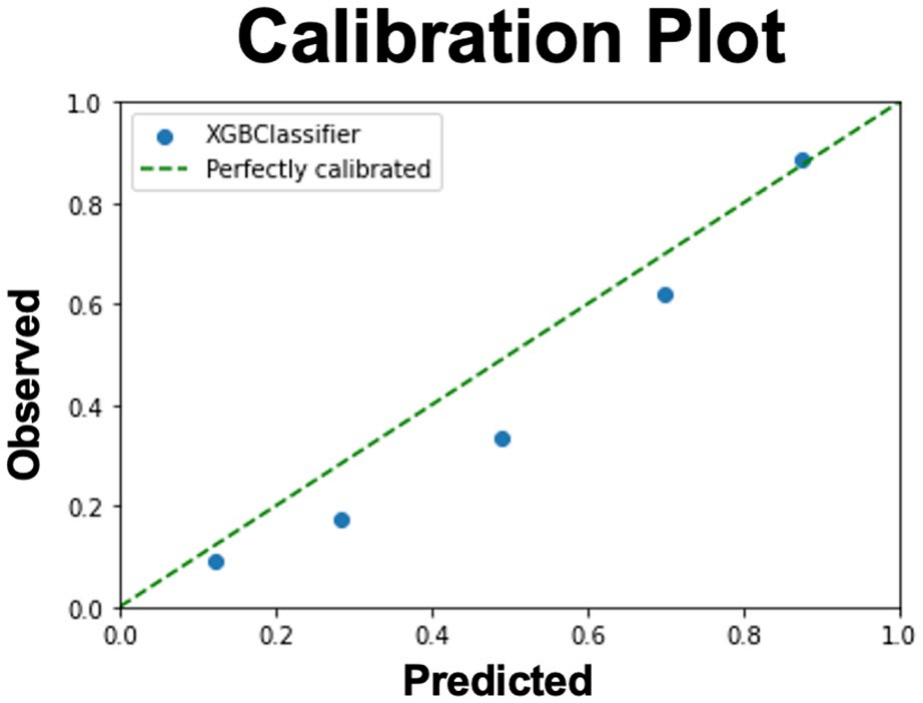
Calibration plot illustrating goodness-of-fit of the XGBoost model tested on the blind test set

Goodness-of-fit of the XGBoost model was visualized with a calibration plot measuring the deciles of predicted probabilities with observed rates (Fig. 3). Supplemental Fig. 1 is the associated histogram corresponding to the calibration plot illustrating the differences in the observed and predicted rates at each probability bucket.

**Fig. 4:**
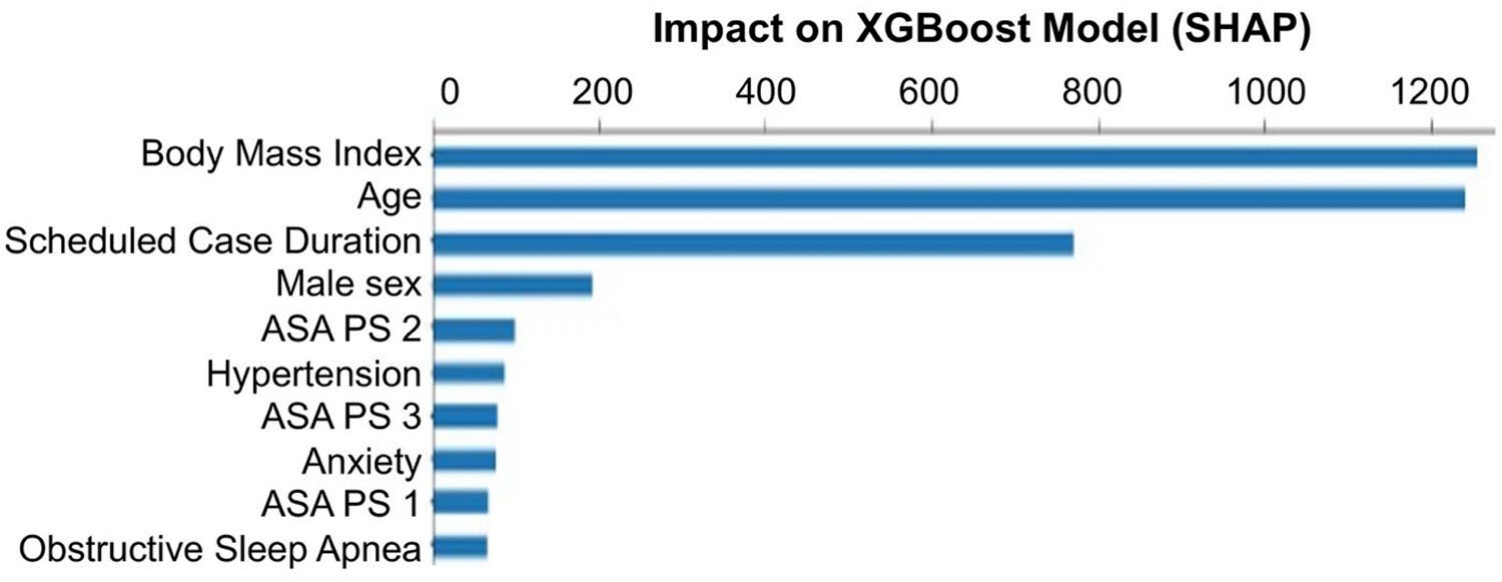
Feature Impact on XGBoost Model as Identified by Shapley Additive Explantations (SHAP)

Features in the XGBoost model identified as having the most significant impact on the model outputs were identified by SHAP analysis (Fig. 4). BMI, age, and scheduled case duration had the highest impact on model performance.

Next, we calculated the prediction of the XGBoost model on each case within the test set – whether that patient would have prolonged or no prolonged PACU LOS. Then we looked at individual operating room days, defined as a full operating room (cases scheduled at least passed 3pm and same surgeon) and resequenced the order of cases based on the prediction calculated from XGBoost (e.g. cases with highest risk of prolonged PACU LOS were scheduled earlier in the day while those with lowest risk were scheduled near the end of the day). There was a total of 198 operating room days analyzed from the test set, in which the median [quartile] number of cases per operating room day was 4 [3, 6] cases. Historically, there were 82 (41.4%) operating room days that had patients stay in the PACU after-hours (passed 7pm). After resequencing of cases based on the machine learning prediction 24 (12.1%) of the operating room days had patients stay in the PACU after-hours (P < 0.0001) (Table 3).

**Table 3:** Improvement in patient discharged after 7pm using machine learning to resequence cases based on predicted prolonged PACU length of stay. An OR day is defined as a full operating room (cases scheduled at least passed 3pm and same surgeon). Machine learning was used to predict cases in the test dataset that would have prolonged PACU length of stay (≥ 180 minutes). Those cases were then moved earlier in the day. Patients with the highest probability of prolonged PACU length of stay were scheduled earliest while those with the lowest probability were scheduled later. Chi-square was used to calculate statistical significance between the categorical outcome

	**Historic Performance**	**Performance Utilizing ML-based Case Resequencing**	**P-value**
Total Number of OR Days	198		
Cases per OR Day, median [quartile]	4 [3, 6]		
# of OR Days Resequenced	n/a	162 (81.8%)	
# OR Days where patient was discharged from PACU after 7pm	82 (41.4%)		<0.0001

*ML* Machine Learning (XGBoost model), *OR* Operating Room, *PACU* Post-Anesthesia Care Unit

## Discussion

Several machine learning models were developed in this study to predict prolonged PACU LOS for outpatient surgeries. The XGBoost model combined with a class balancer, SMOTE, outperformed the other models and was used to identify at-risk patients on a separate test set. Using this knowledge, the surgical procedures were resequenced and re-evaluated, demonstrating a statistically significant reduction in after-hours PACU care. Though previous studies have reported the use of machine learning for PACU LOS prediction [10, 18–20], utilization of ensemble learning with features only known preoperatively and the subsequent testing of the ability of the model to reduce after hours PACU stay is novel. The potential to resequence cases using preoperative metrics could reduce staffing overages and other associated costs.

Running surgical centers incurs various indirect and direct costs [21]. To enhance operational efficiency and patient care, it is crucial to decrease labor costs in outpatient surgery centers. The cost of PACU staffing varies among institutions and is influenced by staffing practices and labor costs, particularly overtime compensation. The number of nurses and anesthesiologists required per patient may also differ among surgery centers. Several studies over the last few decades have evaluated interventions that may reduce costs in recovery rooms - including fast-tracking programs – which aimed to reduce both PACU time and staffing needs [22–24]. While many of these studies have demonstrated decreased PACU stay, it is unclear if total labor costs or workload in an ambulatory surgery setting were significantly reduced.

The concept of case resequencing - which aims to strategically order cases based on predicted PACU stay - has the theoretical benefit of reducing after-hour care in the PACU. A reduction in staffing needs during after-hours may translate to decreased overtime pay for both nursing and anesthesiology but would also depend on the staffing structure at a given institution. To address this issue, a machine learning-based model capable of predicting cases with prolonged PACU stay was developed and then the simulated resequencing of cases from historic data validated the ability of resequencing to reduce PACU LOS using key features from the machine learning models. It is important to point out barriers of implementing this type of clinical decision support in practice [25]. For example, surgeons may not want to lose control over the order of their cases or there may be existing case conflicts that would not allow certain cases to be scheduled at a different time. Nonetheless, the simulation demonstrated nearly a threefold decrease in potential after-hour staffing needs. While it would likely not be possible to re-sequence every operating room in practice due to other conflicts (e.g. surgeon preference, patient requests, equipment conflicts), it may still provide some benefit. A prospective study would be needed to validate the potential effectiveness.

Our model included features that were previously described in other studies evaluating clinical features associated with PACU LOS. We previously reported the use of multivariable logistic regression to predict prolonged PACU LOS after outpatient surgery among over 4,000 patients and included the following features: morbid obesity, hypertension, surgical specialty, primary anesthesia type, and scheduled case duration [10]. Elsharydah et al. reported a subsequent study validating this model on their institutional data and refined a model specific to their institution using similar features, including anesthesia type, obstructive sleep apnea, surgical specialty, and scheduled case duration [20]. Development of a predictive model for prolonged PACU LOS after laparoscopic cholecystectomy had also been reported [19]. The current study showed the advantages of using ensemble learning such as XGBoost and oversampling techniques such as SMOTE to improve prediction. As the models were trained solely with known pre-operative variables independent from anesthesia type, actual surgical duration, and other intraoperative factors, the outputs can be used to suggest an ideal case sequencing order a day prior to surgery. Implementing this technique in a prospective study would be an important next step. The cost-effectiveness of ambulatory surgery has been established in multiple clinical settings, and predictive models that improve efficiency can optimize resource utilization [26–28].

The most impactful features in the XGBoost model were BMI, age, and scheduled case duration. The association of BMI and PACU LOS is controversial as various studies are demonstrated a correlation [10], while others have not [20, 29]. BMI has a strong correlation with obstructive sleep apnea, which is a known risk factor for prolonged PACU LOS, and thus may be a mediator rather than an independent risk factor [20, 29]. Scheduled case duration is also a known predictor for prolonged PACU LOS, which may be a direct indicator of surgical complexity and need for longer anesthesia times [10, 20, 29]. Age has also been demonstrated to be a predictor for prolonged PACU LOS [19], which could be related to longer recovery needed for elderly patients after anesthesia. Of note, our dataset did not include any features that were unknown preoperatively (e.g. final anesthesia type and actual case duration) as the purpose of the model was be able to re-sequence cases prior to day of surgery in an effort to improve PACU staffing efficiency.

This study has several limitations such as its retrospective design, data being collected from a single site, focusing on specific comorbidities to the exclusion of others, and not providing a severity level of comorbidities (i.e. hypertension or obstructive sleep apnea). Subsequent models developed may include additional features not included in this iteration, including history of postoperative nausea and vomiting, concomitant medication use, and cognitive baseline. Moreover, the data was collected from an ambulatory surgery facility at a quaternary academic medical center which may not be representative of the general outpatient population, and many patients in this dataset were ASA ≥ 3. Further research should include validating these models in external settings and conducting a prospective study to evaluate the impact of the model on PACU efficiency.

In conclusion, we described the development of a predictive model using XGBoost and a class balancer to identify ambulatory surgical patients that were highest risk for prolonged PACU stay. This information was then used in simulation that re-sequenced surgeries in historic operating room days. The results demonstrated a statistically significant decrease in the number of patients that stayed passed 7pm in an ambulatory surgery PACU.

### Supplementary Information

Below is the link to the electronic supplementary material.

Supplementary file1 (JPEG 78 KB)

Supplementary file2 (DOCX 45 KB)

## Data Availability

Source code is available in the Supplementary Files.
