# Supplementary figures and images for: Machine Learning Prediction Models to Reduce Length of Stay at Ambulatory Surgery Centers Through Case Resequencing
Source: J Med Syst. 2023 Jul 10;47(1):71. doi: 10.1007/s10916-023-01966-9 (PMC10333394; doi:10.1007/s10916-023-01966-9)

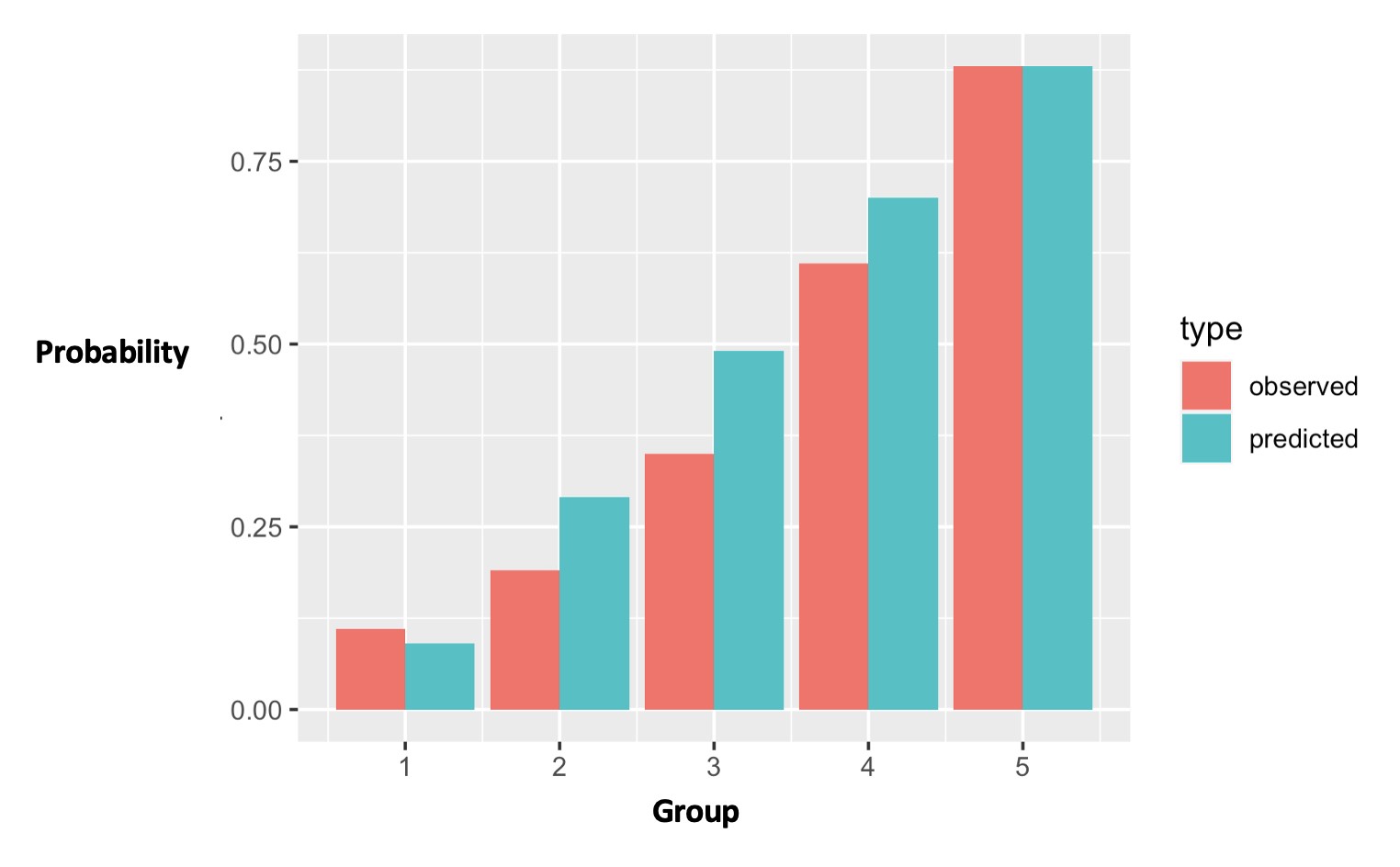

Supplement: Supplementary file 1 — Supplementary file1 (JPEG 78 KB) [file 10916_2023_1966_MOESM1_ESM.jpeg]
