# Supplementary material for: Machine Learning Prediction Models to Reduce Length of Stay at Ambulatory Surgery Centers Through Case Resequencing
Source: J Med Syst. 2023 Jul 10;47(1):71. doi: 10.1007/s10916-023-01966-9 (PMC10333394; doi:10.1007/s10916-023-01966-9)
Supplement: Supplementary file 2 — Supplementary file2 (DOCX 45 KB) [file 10916_2023_1966_MOESM2_ESM.docx]

| **Surgical Procedure Name** | **Frequency** | **Percentage** |
| --- | --- | --- |
| ABDOMINOPLASTY | 10 | 0.092% |
| ABLATION, DYSPLASIA, VULVA, USING CO2 LASER | 34 | 0.311% |
| ABLATION, ENDOMETRIUM, THERMAL | 37 | 0.339% |
| ABLATION, LESION, VAGINA, USING LASER | 2 | 0.018% |
| AMPUTATION, FOOT, FIRST RAY | 3 | 0.027% |
| AMPUTATION, HAND OR FINGER | 12 | 0.110% |
| AMPUTATION, TOE | 3 | 0.027% |
| ANAL DILATION | 4 | 0.037% |
| ANTERIOR COLPORRHAPHY | 1 | 0.009% |
| APPLICATION, FAT GRAFT | 72 | 0.659% |
| APPLICATION, GRAFT, SKIN, FULL-THICKNESS, TO HEAD OR FACE | 2 | 0.018% |
| APPLICATION, GRAFT, SKIN, SPLIT-THICKNESS, TO FACE | 1 | 0.009% |
| APPLICATION, GRAFT, SKIN, SPLIT-THICKNESS, TO TORSO | 1 | 0.009% |
| ARTHRODESIS, FINGER | 35 | 0.320% |
| ARTHROPLASTY, ELBOW | 5 | 0.046% |
| ARTHROPLASTY, FINGER | 16 | 0.146% |
| ARTHROPLASTY, FOOT | 1 | 0.009% |
| ARTHROPLASTY, WRIST | 2 | 0.018% |
| ARTHROSCOPY OR ARTHROTOMY, HIP | 81 | 0.741% |
| ARTHROSCOPY, ANKLE | 22 | 0.201% |
| ARTHROSCOPY, ELBOW | 13 | 0.119% |
| ARTHROSCOPY, KNEE | 223 | 2.041% |
| ARTHROSCOPY, KNEE, WITH MEDIAL PATELLOFEMORAL LIGAMENT REPAIR | 1 | 0.009% |
| ARTHROSCOPY, SHOULDER | 201 | 1.839% |
| ARTHROSCOPY, SHOULDER, WITH ROTATOR CUFF REPAIR | 121 | 1.107% |
| ARTHROSCOPY, SHOULDER, WITH SLAP LESION REPAIR | 15 | 0.137% |
| ARTHROSCOPY, SHOULDER, WITH SUBACROMIAL DECOMPRESSION AND POSSIBLE DISTAL CLAVICLE EXCISION | 3 | 0.027% |
| ARTHROSCOPY, WRIST | 21 | 0.192% |
| ARTHROTOMY, ANKLE | 2 | 0.018% |
| ARTHROTOMY, ELBOW | 10 | 0.092% |
| ARTHROTOMY, KNEE | 5 | 0.046% |
| ARTHROTOMY, SHOULDER | 4 | 0.037% |
| ARTHROTOMY, WRIST, WITH RADIAL STYLOID EXCISION | 3 | 0.027% |
| ASPIRATION OR INJECTION, JOINT, LOWER EXTREMITY | 1 | 0.009% |
| AUGMENTATION, BREAST | 34 | 0.311% |
| AUGMENTATION, BREAST, WITH MASTOPEXY | 1 | 0.009% |
| BALLOON COMPRESSION, NERVE, TRIGEMINAL | 1 | 0.009% |
| BASIC FOOT | 163 | 1.492% |
| BASIC HAND | 19 | 0.174% |
| BIOPSY OR EXCISION, LESION, MOUTH | 3 | 0.027% |
| BIOPSY, ARTERY, TEMPORAL | 3 | 0.027% |
| BIOPSY, BONE | 4 | 0.037% |
| BIOPSY, BREAST | 17 | 0.156% |
| BIOPSY, BREAST AND SENTINEL LYMPH NODE | 20 | 0.183% |
| BIOPSY, BREAST, BILATERAL, WITH LUMPECTOMY | 7 | 0.064% |
| BIOPSY, BREAST, WITH LUMPECTOMY | 189 | 1.730% |
| BIOPSY, CERVIX | 5 | 0.046% |
| BIOPSY, LESION, PENIS | 1 | 0.009% |
| BIOPSY, MUSCLE, LOWER EXTREMITY | 1 | 0.009% |
| BIOPSY, NERVE | 1 | 0.009% |
| BIOPSY, PROSTATE | 4 | 0.037% |
| BIOPSY, PROSTATE, TRANSPERINEAL APPROACH | 99 | 0.906% |
| BIOPSY, RECTAL | 10 | 0.092% |
| BIOPSY, VULVA | 2 | 0.018% |
| BLEPHAROPLASTY, LOWER EYELID | 4 | 0.037% |
| BLEPHAROPLASTY, UPPER EYELID | 12 | 0.110% |
| BLEPHAROPLASTY, WITH BROWLIFT | 3 | 0.027% |
| BRACHYTHERAPY, PROSTATE | 8 | 0.073% |
| BREAST RECONSTRUCTION - STAGE 1 | 1 | 0.009% |
| BURSECTOMY OR SYNOVECTOMY, HAND | 1 | 0.009% |
| BURSECTOMY, ELBOW OLECRANON | 10 | 0.092% |
| CANALOPLASTY, EAR | 20 | 0.183% |
| CAPSULECTOMY OR CAPSULOTOMY, MCP JOINT | 3 | 0.027% |
| CAPSULOTOMY OR CAPSULECTOMY, BREAST | 22 | 0.201% |
| CARPAL TUNNEL RELEASE, ENDOSCOPIC | 19 | 0.174% |
| CARPECTOMY | 12 | 0.110% |
| CHEILECTOMY | 29 | 0.265% |
| CHOLECYSTECTOMY, LAPAROSCOPIC | 111 | 1.016% |
| CIRCUMCISION | 30 | 0.275% |
| CLOSURE, WOUND | 1 | 0.009% |
| CLOSURE, WOUND, USING GLUTEAL OR ABDOMINAL ADVANCEMENT FLAP | 2 | 0.018% |
| COLONOSCOPY | 406 | 3.715% |
| COLPOCLEISIS | 17 | 0.156% |
| COLPOPEXY, VAGINAL EXTRAPERITONEAL APPROACH | 25 | 0.229% |
| COLPORRHAPHY, COMBINED ANTEROPOSTERIOR | 47 | 0.430% |
| COLPORRHAPHY, POSTERIOR | 16 | 0.146% |
| COLPOSCOPY | 24 | 0.220% |
| CONIZATION, CERVIX | 21 | 0.192% |
| CORRECTION, BUNIONETTE | 6 | 0.055% |
| CORRECTION, HALLUX VALGUS | 34 | 0.311% |
| CORRECTION, HAMMER TOE | 12 | 0.110% |
| CREATION, FLAP, FOREHEAD | 1 | 0.009% |
| CREATION, FLAP, ROTATIONAL | 14 | 0.128% |
| CREATION, FLAP, ROTATIONAL, HAND | 4 | 0.037% |
| CRYOABLATION, PROSTATE, TRANSPERINEAL | 14 | 0.128% |
| CYSTOSCOPY | 134 | 1.226% |
| CYSTOSCOPY, W/ SUPRAPUBIC TUBE PLACEMENT, PERCUTANEOUS | 3 | 0.027% |
| CYSTOSCOPY, WITH BIOPSY | 9 | 0.082% |
| CYSTOSCOPY, WITH RETROGRADE PYELOGRAM | 4 | 0.037% |
| CYSTOSCOPY, WITH RETROGRADE URETHROGRAM | 1 | 0.009% |
| CYSTOSCOPY, WITH TRANSURETHRAL INJECTION | 51 | 0.467% |
| CYSTOSCOPY, WITH TRANSURETHRAL NEEDLE ABLATION OF PROSTATE | 40 | 0.366% |
| CYSTOSCOPY, WITH URETERAL STENT INSERTION | 13 | 0.119% |
| CYSTOSTOMY, SUPRAPUBIC | 13 | 0.119% |
| CYSTOURETHROSCOPY, WITH DIRECT VISION INTERNAL URETHROTOMY | 21 | 0.192% |
| DACRYOCYSTORHINOSTOMY | 2 | 0.018% |
| DEBRIDEMENT, OSTEOMYELITIS, FOOT OR ANKLE | 2 | 0.018% |
| DEBRIDEMENT, SHOULDER, ARTHROSCOPIC | 1 | 0.009% |
| DEBRIDEMENT, TORSO | 1 | 0.009% |
| DEBRIDEMENT, WOUND | 3 | 0.027% |
| DEBRIDEMENT, WOUND OR ULCER, FOOT OR ANKLE | 8 | 0.073% |
| DECOMPRESSION, NERVE | 2 | 0.018% |
| DECOMPRESSION, NERVE, UPPER EXTREMITY | 3 | 0.027% |
| DECOMPRESSION, ORBIT | 4 | 0.037% |
| DECOMPRESSION, ULNAR NERVE | 37 | 0.339% |
| DILATION AND CURETTAGE (OB) | 5 | 0.046% |
| DILATION AND CURETTAGE, UTERUS | 230 | 2.105% |
| DILATION AND EVACUATION, UTERUS | 68 | 0.622% |
| DILATION, ESOPHAGUS | 2 | 0.018% |
| DILATION, EUSTACHIAN TUBE, USING BALLOON | 1 | 0.009% |
| DISPLACEMENT, VOCAL CORD, WITH BIOLOGICAL GRAFT | 1 | 0.009% |
| DISSECTION, LYMPH NODE, AXILLARY | 6 | 0.055% |
| DISSECTION, LYMPH NODE, SENTINEL | 30 | 0.275% |
| DISSECTION, NECK | 4 | 0.037% |
| EGD, INTRAOPERATIVE | 124 | 1.135% |
| ELECTROEJACULATION | 6 | 0.055% |
| ENDOPYELOTOMY, RETROGRADE | 1 | 0.009% |
| ENDOSCOPY, POUCH, INTESTINE | 3 | 0.027% |
| ESOPHAGOSCOPY | 2 | 0.018% |
| ESOPHAGOSCOPY, WITH DILATION | 4 | 0.037% |
| EXAM UNDER ANESTHESIA, ANORECTAL | 178 | 1.629% |
| EXAM UNDER ANESTHESIA, PELVIS | 1 | 0.009% |
| EXAM UNDER ANESTHESIA, VAGINA | 59 | 0.540% |
| EXCISION OR CURETTAGE, BONE LESION, HUMERUS, PROXIMAL | 1 | 0.009% |
| EXCISION OR CURETTAGE, BONE LESION, TARSAL OR METATARSAL | 1 | 0.009% |
| EXCISION OR CURETTAGE, NEOPLASM, RADIUS, DISTAL | 2 | 0.018% |
| EXCISION, ANAL SKIN TAG | 4 | 0.037% |
| EXCISION, BONE, UPPER EXTREMITY | 4 | 0.037% |
| EXCISION, BOSS, FOOT | 1 | 0.009% |
| EXCISION, CONDYLOMA | 57 | 0.522% |
| EXCISION, CYST | 31 | 0.284% |
| EXCISION, CYST, BONE, HAND | 2 | 0.018% |
| EXCISION, CYST, OVARY, LAPAROSCOPIC | 68 | 0.622% |
| EXCISION, CYST, VAGINA | 1 | 0.009% |
| EXCISION, DIVERTICULUM, URETHRAL | 8 | 0.073% |
| EXCISION, EXOSTOSIS, EXTERNAL AUDITORY CANAL | 1 | 0.009% |
| EXCISION, GYNECOMASTIA | 1 | 0.009% |
| EXCISION, HIDRADENITIS | 2 | 0.018% |
| EXCISION, HYDROCELE | 13 | 0.119% |
| EXCISION, LESION / REVISION, SCAR/ WOUND CLOSURE, HEAD / NECK / FACE | 1 | 0.009% |
| EXCISION, LESION OR FOREIGN BODY, HEAD OR NECK | 3 | 0.027% |
| EXCISION, LESION, HEAD OR NECK | 133 | 1.217% |
| EXCISION, LESION, LOWER EXTREMITY | 31 | 0.284% |
| EXCISION, LESION, TORSO | 44 | 0.403% |
| EXCISION, LESION, UPPER EXTREMITY | 200 | 1.830% |
| EXCISION, LESION, VULVA | 27 | 0.247% |
| EXCISION, LYMPH NODE, CERVICAL | 14 | 0.128% |
| EXCISION, MANDIBLE | 3 | 0.027% |
| EXCISION, MORTON'S NEUROMA | 2 | 0.018% |
| EXCISION, NAIL AND NAIL MATRIX | 5 | 0.046% |
| EXCISION, NEOPLASM, FINGER | 38 | 0.348% |
| EXCISION, NEOPLASM, PAROTID GLAND | 22 | 0.201% |
| EXCISION, NEOPLASM, SOFT TISSUE, HUMERUS OR ELBOW | 1 | 0.009% |
| EXCISION, NEOPLASM, SOFT TISSUE, SHOULDER | 1 | 0.009% |
| EXCISION, NEOPLASM, SUBFASCIAL OR INTRAMUSCULAR, FOREARM OR WRIST | 1 | 0.009% |
| EXCISION, NEUROFIBROMA OR NEUROLEMMOMA, MAJOR PERIPHERAL NERVE | 1 | 0.009% |
| EXCISION, PILONIDAL CYST | 22 | 0.201% |
| EXCISION, RADIUS, HEAD AND NECK REGION, PARTIAL | 1 | 0.009% |
| EXCISION, SALIVARY GLAND | 9 | 0.082% |
| EXCISION, SCAR | 7 | 0.064% |
| EXCISION, SPERMATOCELE | 2 | 0.018% |
| EXCISION, VARICOCELE | 36 | 0.329% |
| EXPLORATION, NERVE, ACOUSTIC | 2 | 0.018% |
| EXPLORATION, SCROTUM | 10 | 0.092% |
| EXPLORATION, WITH NERVE REPAIR OF HAND OR WRIST | 18 | 0.165% |
| EXTRACORPOREAL SHOCKWAVE LITHOTRIPSY (ESWL) | 81 | 0.741% |
| FACELIFT | 2 | 0.018% |
| FACELIFT, WITH BROWLIFT | 2 | 0.018% |
| FACIAL RECONSTRUCTION, FASCIA LATA STATIC SUSPENSION | 1 | 0.009% |
| FAT GRAFT, FACE | 2 | 0.018% |
| FISTULECTOMY, ANAL | 205 | 1.876% |
| FISTULOTOMY, ANAL | 6 | 0.055% |
| FLAP PROCEDURE, MUSCLE, MYOCUTANEOUS, OR FASCIOCUTANEOUS, UPPER EXTREMITY | 1 | 0.009% |
| FOOT AND ANKLE - ARTHRODESIS | 12 | 0.110% |
| FOOT AND ANKLE - ARTHROSCOPY/OLT | 5 | 0.046% |
| FOOT AND ANKLE - FOREFOOT | 8 | 0.073% |
| FOOT AND ANKLE - FRACTURES/TRAUMA | 6 | 0.055% |
| FOOT AND ANKLE - HALLUX VALGUS | 11 | 0.101% |
| FOOT AND ANKLE - INFECTION AND AMPUTATION | 10 | 0.092% |
| FOOT AND ANKLE - MASS OR FOREIGN BODY EXCISION | 10 | 0.092% |
| FOOT AND ANKLE - REPAIR/RECONSTRUCTION | 32 | 0.293% |
| FREE FLAP, RADIAL, FASCIOCUTANEOUS, WITH MICROVASC ANASTOMOSIS | 1 | 0.009% |
| FUSION, CAPITATE, HAMATE, TRIQUETRUM, AND LUNATE BONES | 9 | 0.082% |
| FUSION, JOINT, ANKLE | 22 | 0.201% |
| FUSION, JOINT, FOOT | 40 | 0.366% |
| FUSION, JOINT, TARSOMETATARSAL | 8 | 0.073% |
| FUSION, JOINT, WRIST | 15 | 0.137% |
| FUSION, SHOULDER | 1 | 0.009% |
| FUSION, TOE | 16 | 0.146% |
| GASTROJEJUNOSTOMY, PECUTANEOUS, ENDOSCOPIC (PEG-J) | 4 | 0.037% |
| GASTROSTOMY, PERCUTANEOUS, ENDOSCOPIC | 1 | 0.009% |
| GLOSSECTOMY | 5 | 0.046% |
| HEMORRHOIDECTOMY | 102 | 0.933% |
| HERNIORRHAPHY, INGUINAL | 45 | 0.412% |
| HYMENECTOMY | 3 | 0.027% |
| HYSTERECTOMY, ABDOMINAL, LAPAROSCOPIC | 5 | 0.046% |
| HYSTERECTOMY, VAGINAL | 10 | 0.092% |
| HYSTERECTOMY, VAGINAL WITH UTEROSACRAL, APR PLUS MIDURETHRAL SLING | 2 | 0.018% |
| HYSTERECTOMY, VAGINAL, LAPAROSCOPIC-ASSISTED | 25 | 0.229% |
| HYSTERECTOMY, VAGINAL, WITH COMBINED ANTEROPOSTERIOR COLPORRHAPHY AND UTEROSACRAL LIGAMENT VAULT SUSPENSION | 6 | 0.055% |
| HYSTEROSCOPY | 208 | 1.903% |
| HYSTEROSCOPY, WITH BIOPSY OR POLYPECTOMY | 471 | 4.310% |
| IMPLANTATION, ALLOGRAFT, KNEE, ARTHROSCOPIC | 1 | 0.009% |
| IMPLANTATION, HEARING AID, BONE ANCHORED | 9 | 0.082% |
| IMPLANTATION, MORPHINE PUMP | 3 | 0.027% |
| IMPLANTATION, PERIPHERAL NERVE STIMULATOR | 2 | 0.018% |
| IMPLANTATION, PROSTHESIS, COCHLEAR | 36 | 0.329% |
| IMPLANTATION, URETHRAL LIFT DEVICE, TRANSPROSTATIC, CYSTOSCOPIC (UROLIFT) | 8 | 0.073% |
| INACTIVE - TRANSURETHRAL DESTRUCTION OF PROSTATE TISSUE, RADIOFREQUENCY THERMOTHERAPY | 1 | 0.009% |
| INCISION AND DRAINAGE, ABSCESS, RECTUM | 13 | 0.119% |
| INCISION AND DRAINAGE, HEAD AND NECK | 5 | 0.046% |
| INCISION AND DRAINAGE, HIP | 2 | 0.018% |
| INCISION AND DRAINAGE, LOWER EXTREMITY | 1 | 0.009% |
| INCISION AND DRAINAGE, TORSO | 6 | 0.055% |
| INCISION AND DRAINAGE, UPPER EXTREMITY | 16 | 0.146% |
| INGUINAL SENTINEL LYMPH NODE MAPPING AND/OR DISSECTION | 1 | 0.009% |
| INJECTION, BOTOX, URETHRA, CYSTOSCOPIC | 2 | 0.018% |
| INJECTION, BOTULINUM TOXIN, ANAL SPHINCTER | 5 | 0.046% |
| INJECTION, MACROPLASTIQUE, URETHRA, CYSTOSCOPIC | 9 | 0.082% |
| INJECTION, SMALL OR INTERMEDIATE JOINT | 10 | 0.092% |
| INSERT OR REPLACE SPINAL NEUROSTIMULATOR GENERATOR OR RECEIVER | 1 | 0.009% |
| INSERTION OR REMOVAL, INFUSION PORT | 3 | 0.027% |
| INSERTION OR REMOVAL, STENT, URETER | 36 | 0.329% |
| INSERTION, ARTIFICIAL SPHINCTER, URINARY | 1 | 0.009% |
| INSERTION, IMPLANT, BREAST, DELAYED, AFTER BREAST SURGERY | 1 | 0.009% |
| INSERTION, IMPLANTABLE PULSE GENERATOR AND ELECTRODES OF SACRAL NERVE STIMULATOR | 2 | 0.018% |
| INSERTION, INTRATHECAL BACLOFEN PUMP | 2 | 0.018% |
| INSERTION, NEUROSTIMULATOR, HYPOGLOSSAL | 2 | 0.018% |
| INSERTION, NEUROSTIMULATOR, SACRAL | 59 | 0.540% |
| INSERTION, PROSTHESIS, ORBIT | 2 | 0.018% |
| INSERTION, PROSTHESIS, TESTICULAR | 2 | 0.018% |
| INSERTION, SLING, MIDURETHRAL | 101 | 0.924% |
| INSERTION, SPINAL CORD STIMULATOR, DORSAL COLUMN | 71 | 0.650% |
| INSERTION, TISSUE EXPANDER | 3 | 0.027% |
| INSERTION, TISSUE EXPANDER, BREAST | 7 | 0.064% |
| INSERTION, TISSUE EXPANDER, BREAST, BILATERAL | 5 | 0.046% |
| INSERTION, VAGUS NERVE STIMULATOR | 4 | 0.037% |
| INSERTION, WEIGHT, UPPER EYELID | 1 | 0.009% |
| IRRIGATION AND DEBRIDEMENT, FOOT | 5 | 0.046% |
| LAPAROSCOPY, DIAGNOSTIC | 113 | 1.034% |
| LARYNGOSCOPY | 47 | 0.430% |
| LARYNGOSCOPY, DIRECT | 41 | 0.375% |
| LARYNGOSCOPY, DIRECT, WITH NEOPLASM EXCISION | 1 | 0.009% |
| LARYNGOSCOPY, WITH BRONCHOSCOPY AND ESOPHAGOSCOPY IF INDICATED | 21 | 0.192% |
| LEEP PROCEDURE | 16 | 0.146% |
| LENGTHENING, ACHILLES TENDON | 2 | 0.018% |
| LENGTHENING, MUSCLE, GASTROCNEMIUS | 9 | 0.082% |
| LIFT PROCEDURE | 6 | 0.055% |
| LIGATION AND STRIPPING, VARICOSE VEINS, OPEN, USING STAB PHLEBECTOMY TECHNIQUE | 21 | 0.192% |
| LIGATION, FALLOPIAN TUBE, BILATERAL | 11 | 0.101% |
| LIPOSUCTION, THIGH | 4 | 0.037% |
| LIPOSUCTION, TRUNK | 48 | 0.439% |
| LIPOSUCTION, ULTRASONIC | 2 | 0.018% |
| LITHOLAPAXY | 22 | 0.201% |
| LUMPECTOMY OR PARTIAL MASTECTOMY, WITH SENTINEL NODE BIOPSY | 102 | 0.933% |
| MAMMOPLASTY, REDUCTION | 59 | 0.540% |
| MANIPULATION, JOINT, SHOULDER OR KNEE, WITH ANESTHESIA | 2 | 0.018% |
| MARSUPIALIZATION, CYST, BARTHOLIN'S GLAND | 6 | 0.055% |
| MASTECTOMY, SIMPLE | 4 | 0.037% |
| MASTECTOMY, SIMPLE, BILATERAL | 4 | 0.037% |
| MASTECTOMY, WITH IMMEDIATE IMPLANT INSERTION | 1 | 0.009% |
| MASTOIDECTOMY | 6 | 0.055% |
| MASTOPEXY | 40 | 0.366% |
| MASTOPEXY, BILATERAL | 11 | 0.101% |
| MENISCECTOMY, KNEE, ARTHROSCOPIC | 2 | 0.018% |
| MICROLARYNGOSCOPY, DIRECT | 1 | 0.009% |
| MYOMECTOMY | 12 | 0.110% |
| MYOMECTOMY, LAPAROSCOPIC | 103 | 0.943% |
| MYOMECTOMY, UTERUS, HYSTEROSCOPIC | 21 | 0.192% |
| NEPHROLITHOTOMY, PERCUTANEOUS, OR PERCUTANEOUS NEPHROLITHOTRIPSY | 1 | 0.009% |
| OOPHORECTOMY, LAPAROSCOPIC | 3 | 0.027% |
| ORCHIECTOMY | 6 | 0.055% |
| ORCHIECTOMY, RADICAL | 3 | 0.027% |
| ORCHIOPEXY | 1 | 0.009% |
| ORIF, ANKLE | 1 | 0.009% |
| ORIF, ELBOW | 11 | 0.101% |
| ORIF, FRACTURE, ANKLE, MAISONNEUVE | 3 | 0.027% |
| ORIF, FRACTURE, CALCANEUS | 1 | 0.009% |
| ORIF, FRACTURE, CLAVICLE | 5 | 0.046% |
| ORIF, FRACTURE, FACIAL BONE | 1 | 0.009% |
| ORIF, FRACTURE, HUMERUS | 5 | 0.046% |
| ORIF, FRACTURE, MANDIBLE, WITH MAXILLOMANDIBULAR FIXATION | 2 | 0.018% |
| ORIF, FRACTURE, METATARSAL BONE OR PHALANX, FOOT | 2 | 0.018% |
| ORIF, FRACTURE, PATELLA | 1 | 0.009% |
| ORIF, FRACTURE, RADIUS AND ULNA, SHAFTS | 7 | 0.064% |
| ORIF, FRACTURE, RADIUS, DISTAL | 109 | 0.997% |
| ORIF, FRACTURE, RADIUS, HEAD | 8 | 0.073% |
| ORIF, FRACTURE, SCAPHOID | 16 | 0.146% |
| ORIF, HAND | 74 | 0.677% |
| OSTEOPLASTY, RADIUS OR ULNA, FOR SHORTENING | 4 | 0.037% |
| OSTEOTOMY, FOOT | 1 | 0.009% |
| OSTEOTOMY, METACARPAL BONE | 2 | 0.018% |
| OSTEOTOMY, PHALANX | 1 | 0.009% |
| OSTEOTOMY, RADIUS, DISTAL THIRD | 2 | 0.018% |
| OSTEOTOMY, TARSAL BONE | 1 | 0.009% |
| PAIN DESTRUCTION INTRAOSSEOUS BASIVERTEBRAL NERVE LUMBAR/SACRUM 1ST 2 VERTEBRAL BODIES | 5 | 0.046% |
| PANNICULECTOMY AND/OR MONSECTOMY | 2 | 0.018% |
| PARATHYROIDECTOMY | 57 | 0.522% |
| PENILE PLICATION | 8 | 0.073% |
| PINNING, UPPER EXTREMITY, PERCUTANEOUS | 1 | 0.009% |
| PLACEMENT, DEEP BRAIN STIMULATOR, STAGE TWO | 9 | 0.082% |
| PLACEMENT, NEUROSTIMULATOR BATTERY | 1 | 0.009% |
| PLASTIC REPAIR, CLEFT PALATE | 1 | 0.009% |
| PYELOGRAM, RETROGRADE | 17 | 0.156% |
| RADICAL RESECTION, NEOPLASM, MALIGNANT, FOOT | 1 | 0.009% |
| RADIOFREQUENCY ABLATION, VARICOSE VEIN | 77 | 0.705% |
| RECONSTRUCTION, BREAST, USING FAT GRAFT | 2 | 0.018% |
| RECONSTRUCTION, BREAST, WITH IMMEDIATE PERMANENT BREAST IMPLANT | 1 | 0.009% |
| RECONSTRUCTION, HAND | 4 | 0.037% |
| RECONSTRUCTION, LIGAMENT, ULNAR COLLATERAL | 1 | 0.009% |
| RECONSTRUCTION, LIGAMENT, WITH TENDON INTERPOSITION | 87 | 0.796% |
| RECONSTRUCTION, NIPPLE | 24 | 0.220% |
| RECONSTRUCTION, NOSE, TIP | 3 | 0.027% |
| REDUCTION, FRACTURE, NASAL BONE, CLOSED | 3 | 0.027% |
| REINSERTION, TENDON, BICEPS, DISTAL, RUPTURED, WITH TENDON GRAFT | 37 | 0.339% |
| RELEASE, CARPAL TUNNEL | 471 | 4.310% |
| RELEASE, CONTRACTURE, HAND, FROM EPIDERMOLYSIS BULLOSA | 26 | 0.238% |
| RELEASE, CONTRACTURE, HEAD AND NECK REGION | 1 | 0.009% |
| RELEASE, DUPUYTREN'S CONTRACTURE | 41 | 0.375% |
| RELEASE, HAND FOR DEQUERVAIN'S TENOSYNOVITIS | 10 | 0.092% |
| RELEASE, TARSAL TUNNEL | 2 | 0.018% |
| RELEASE, TRIGGER FINGER | 218 | 1.995% |
| REMOVAL OF INTERMAXILLARY FIXATION BARS AND WIRES (ARCH BAR REMOVAL) | 5 | 0.046% |
| REMOVAL OR REVISION, SLING, MIDURETHRAL | 16 | 0.146% |
| REMOVAL, CALCULUS, URETER, URETEROSCOPIC | 83 | 0.760% |
| REMOVAL, FOREIGN BODY, INSIDE OF MOUTH | 1 | 0.009% |
| REMOVAL, FOREIGN BODY, LOWER EXTREMITY | 3 | 0.027% |
| REMOVAL, HARDWARE, LOWER EXTREMITY | 49 | 0.448% |
| REMOVAL, IMPLANT, BREAST | 42 | 0.384% |
| REMOVAL, IMPLANT, BREAST, WITH MASTOPEXY | 1 | 0.009% |
| REMOVAL, NEUROSTIMULATOR ELECTRODES, SACRAL | 1 | 0.009% |
| REMOVAL, NEUROSTIMULATOR, SACRAL | 1 | 0.009% |
| REMOVAL, ORBITAL IMPLANT | 1 | 0.009% |
| REMOVAL, ORTHOPEDIC HARDWARE, UPPER EXTREMITY | 100 | 0.915% |
| REMOVAL, STENT, URETER, CYSTOSCOPIC | 4 | 0.037% |
| REMOVAL, TISSUE EXPANDER, BREAST | 3 | 0.027% |
| REMOVAL, VAGINAL WALL, PARTIAL | 5 | 0.046% |
| REPAIR OF NONUNION HUMERUS FRACTURE | 1 | 0.009% |
| REPAIR, ACL AND PCL, ARTHROSCOPIC ASSISTED | 158 | 1.446% |
| REPAIR, ANKLE, BROSTROM-GOULD | 4 | 0.037% |
| REPAIR, BLOWOUT FRACTURE, ORBIT | 5 | 0.046% |
| REPAIR, FRACTURE NONUNION, BONE | 5 | 0.046% |
| REPAIR, FRACTURE, ORBIT | 2 | 0.018% |
| REPAIR, HAMSTRING, PROXIMAL | 7 | 0.064% |
| REPAIR, HERNIA, INCISIONAL | 4 | 0.037% |
| REPAIR, HERNIA, INGUINAL, BILATERAL, LAPAROSCOPIC | 25 | 0.229% |
| REPAIR, HERNIA, INGUINAL, LAPAROSCOPIC | 193 | 1.766% |
| REPAIR, HERNIA, UMBILICAL | 86 | 0.787% |
| REPAIR, HERNIA, UMBILICAL, LAPAROSCOPIC | 11 | 0.101% |
| REPAIR, HERNIA, VENTRAL | 12 | 0.110% |
| REPAIR, KNEE, POSTEROLATERAL CORNER | 6 | 0.055% |
| REPAIR, LIGAMENT, COLLATERAL, FINGER OR HAND | 15 | 0.137% |
| REPAIR, MOHS PROCEDURE DEFECT, HEAD OR NECK | 3 | 0.027% |
| REPAIR, NERVE, PERONEAL | 2 | 0.018% |
| REPAIR, QUADRICEPS | 1 | 0.009% |
| REPAIR, TENDON OR LIGAMENT, HAND OR WRIST | 51 | 0.467% |
| REPAIR, TENDON, ACHILLES | 12 | 0.110% |
| REPAIR, TENDON, ANKLE OR FOOT | 1 | 0.009% |
| REPAIR, TENDON, PATELLA | 4 | 0.037% |
| REPAIR, TENDON, PERONEAL | 3 | 0.027% |
| REPAIR, TENDON, UPPER EXTREMITY | 25 | 0.229% |
| REPAIR, VENTRAL HERNIA, LAPAROSCOPIC | 3 | 0.027% |
| REPLACEMENT OF TISSUE EXPANDER | 27 | 0.247% |
| REPLACEMENT, IMPLANT, BREAST | 4 | 0.037% |
| REPLACEMENT, NEUROSTIMULATOR BATTERY | 9 | 0.082% |
| RESECTION, BLADDER TUMOR, TRANSURETHRAL | 53 | 0.485% |
| RESECTION, BLADDER TUMOR, TRANSURETHRAL, WITH INSTILLATION MITOMYCIN C | 1 | 0.009% |
| RESECTION, INTRAUTERINE SEPTUM, HYSTEROSCOPIC | 1 | 0.009% |
| RESECTION, PROSTATE, TRANSURETHRAL | 3 | 0.027% |
| REVISION OR REMOVAL OF NEUROSTIM PULSE GENERATOR OR RECEIVER | 6 | 0.055% |
| REVISION OR REMOVAL, MESH, VAGINA | 1 | 0.009% |
| REVISION, RECONSTRUCTION, BREAST | 38 | 0.348% |
| RHINOPLASTY | 18 | 0.165% |
| RHINOPLASTY/ SEPTOPLASTY | 102 | 0.933% |
| SACROSPINOUS LIGAMENT VAGINAL VAULT SUSPENSION | 9 | 0.082% |
| SACROSPINOUS LIGAMENT VAGINAL VAULT SUSPENSION PLUS MIDURETHRAL SLING | 2 | 0.018% |
| SALPINGECTOMY, LAPAROSCOPIC | 13 | 0.119% |
| SALPINGOOPHORECTOMY, LAPAROSCOPIC | 50 | 0.458% |
| SEPTOPLASTY, WITH TURBINATE REDUCTION | 122 | 1.116% |
| SEPTOPLASTY, WITH TURBINATE REDUCTION AND RHINOPLASTY | 1 | 0.009% |
| SIALENDOSCOPY, WITH SIALODOCHOPLASTY IF INDICATED | 37 | 0.339% |
| SIGMOIDOSCOPY, FLEXIBLE | 26 | 0.238% |
| SIGMOIDOSCOPY, RIGID, WITH BIOPSY | 20 | 0.183% |
| SINUS SURGERY ENDOSCOPIC LIMITED W/ IMAGE GUIDANCE | 103 | 0.943% |
| SINUS SURGERY, ENDOSCOPIC | 116 | 1.061% |
| SINUS SURGERY, ENDOSCOPIC, WITH IMAGING GUIDANCE | 314 | 2.873% |
| SKIN GRAFTING; NON-BURN | 7 | 0.064% |
| SLING, PUBOVAGINAL | 3 | 0.027% |
| STABILIZATION, ANKLE | 1 | 0.009% |
| STAPEDECTOMY, OR STAPEDECTOMY REVISION | 47 | 0.430% |
| SUPERFICIALIZATION, AV FISTULA | 1 | 0.009% |
| SURGICAL PROCUREMENT AND TRANSFER, NERVE, PERIPHERAL | 4 | 0.037% |
| SUSPENSION, NASAL VALVE | 5 | 0.046% |
| TENDON RECONSTRUCTION, POSTERIOR TIBIAL, WITH ACCESSORY NAVICULAR BONE EXCISION | 1 | 0.009% |
| TENOLYSIS, FLEXOR TENDON, PALM AND FINGER, EACH TENDON | 7 | 0.064% |
| TENOSYNOVECTOMY, FOREARM OR WRIST | 9 | 0.082% |
| TENOSYNOVECTOMY, HAND | 5 | 0.046% |
| THYROIDECTOMY | 42 | 0.384% |
| TONSILLECTOMY AND ADENOIDECTOMY | 60 | 0.549% |
| TRACHEOBRONCHOSCOPY | 2 | 0.018% |
| TRANSANAL HEMORRHOIDAL DEARTERIALIZATION | 3 | 0.027% |
| TRANSPOSITION, NERVE, LOWER EXTREMITY | 1 | 0.009% |
| TRANSPOSITION, ULNAR NERVE | 34 | 0.311% |
| TUBAL LIGATION, LAPAROSCOPIC | 41 | 0.375% |
| TYMPANOPLASTY | 42 | 0.384% |
| TYMPANOPLASTY WITH MASTOIDECTOMY | 64 | 0.586% |
| TYMPANOTOMY WITH INTUBATION | 6 | 0.055% |
| ULTRASOUND GUIDED EXTRACORPOREAL LITHOTRIPSY | 1 | 0.009% |
| URETEROSCOPY, NON-STONE | 93 | 0.851% |
| URETEROSCOPY, STONE | 26 | 0.238% |
| URETHROPLASTY | 3 | 0.027% |
| URETHROPLASTY, WITH BUCCAL MUCOSAL GRAFT | 3 | 0.027% |
| URETHROSTOMY, PERINEAL | 1 | 0.009% |
| UVULOPALATOPHARYNGOPLASTY (UPPP) | 18 | 0.165% |
| VAPORIZATION, PROSTATE, PHOTOSELECTIVE, USING 532NM GREENLIGHT LASER | 4 | 0.037% |
| VASECTOMY | 26 | 0.238% |
| TOTAL | 10928 | 100.000% |
